# Quality Control of Natural Product Medicine and Nutrient Supplements 2014

**DOI:** 10.1155/2014/109068

**Published:** 2014-08-21

**Authors:** Ying-Yong Zhao, Shuang-Qing Zhang, Feng Wei, Yu-Ming Fan, Feng Sun, Shuhua Bai

**Affiliations:** ^1^Key Laboratory of Resource Biology and Biotechnology in Western China, Ministry of Education, Department of Traditional Chinese Medicine, College of Life Sciences, Northwest University, No. 229 Taibai North Road, Xi'an, Shaanxi 710069, China; ^2^Department of Nutrition and Metabolism, National Institute for Nutrition and Food Safety, China Center for Disease Control and Prevention, 27 Nanwei Road, Xicheng District, Beijing 100050, China; ^3^Department of Traditional Chinese Medicine and Ethnic Medicine, National Institutes for Food and Drug Control, State Food and Drug Administration, 2 Tiantan Xili, Beijing 100050, China; ^4^National Institutes for Food and Drug Control, State Food and Drug Administration, 2 Tiantan Xili, Beijing 100050, China; ^5^Department of Obstetrics & Gynaecology, Yong Loo Lin School of Medicine, National University of Singapore, 10 Medical Drive, Singapore 117597; ^6^Department of Basic Pharmaceutical Sciences, School of Pharmacy, Husson University, 1 College Circle, Bangor, ME 04401, USA

As one of the oldest remedies, the natural medicine products and nutrient supplements have been broadly used to prevent and treat various human diseases for thousands of years. Ginkgo (*Ginkgo biloba*) could treat circulatory disorders and enhance memory, kava-kava (*Piper methysticum*) could elevate mood and produce a feeling of relaxation, valerian (*Valeriana officinalis*) could help in sleep, and the list could go on [1]. Possibly recommended by medical practitioners, consumers consider natural products as an alternative to decrease health cost, alleviate self-diagnosed diseases, or supplement prescribed therapeutic regimens. The natural products have become a large sale on a global basis and they represent an increasing share in healthcare market reaching estimated $6 billion annually only in the USA [2, 3].

Labeled as “natural,” the natural medications and nutrition supplements often puzzle consumers turning them to “completely safe” products based on the perception. In fact, many natural products affect the body in unwanted ways that can potentially lead to serious side effects, even life-threatening results, because many products contain unknown and unquantified active ingredients [4, 5]. It is very important to promote a full and open discussion on quality control of nature medicine products and nutrient supplements for consumers and professionals. Actually, studies on natural products are challenging by several reasons [6–9]. As nature products are plant-driven, product variation can occur at multiple stages of production, from season variation and identification of plant source to the manufacturing process. More importantly, unlike most modern medications, natural products are typical mixtures that vary greatly. The identity and quantity of the compounds contained within a given natural product is a frequent complication of studies [6]. Even when composition is relatively well understood, the lack of simple and effective analytical methods hampers the ability to extrapolate results beyond quality studied [8]. Furthermore, pharmacological effects of many components in the natural products are still not clear due, in large part, to deficiency of a number of fully developed systems of investigation and predication [9].

This special issue has been driven from the editors' hope that the readers will be able to obtain different kinds of knowledge on natural products from many contributors. Ten publications from original research are encompassing methods and techniques relevant to the detection, separation, purification, identification, and quantification of compounds in biochemistry, cellular and molecular biology, and biotechnology fields. The authors of this thematic issue are researchers from a wide variety of areas and provide a comprehensive summary of the most recent knowledge and references on quality control of natural products.

From an analytical perspective, this focus issue provides a forum for discussion among researchers engaged in basic scientific work in natural product chemistry. As extraction procedures and ingredients of natural products are a permanently demanding challenge for the analytical chemist, highly efficient techniques to handle the problems associated with sample preparation, separation, and identification have been applied to reduce the complexity of plant materials in this issue. With the development of stationary phases allowing endless possibilities in terms of selectivity, analytical platforms including long-term enhanced ultraviolet-B (UV-B) spectroscopy, FI-NIR based imaging, doubly charged selected ion coupled with mass spectrometry, and liquid chromatography-ultraviolet detection coupled with electrospray ionization quadrupole time-of-flight mass spectrometry (LC-UV-ESI-Q/TOF/MS) have been established to profile and characterize plant materials and/or their extractives. Vibrational spectroscopies offer the advantages of fast, noninvasive, and simultaneous determination of chemical and physical properties being suitable for high-throughput analytical quality control.

From a drug development perspective, this issue provides a forum for scientific research expanding to preclinical studies. Novel approaches have been developed for screening the prototype components and* in vivo* metabolites based on rapid and sensitive techniques. The identification and structural elucidation of the chemical compounds provide essential data for further pharmacological and pharmacokinetic studies. Moreover, two articles are devoted to studying pharmacological effects of natural products. The complexity of single medication or compound preparations has had effect on biological signal transductions in animal models and those studies could provide key information for further exploration. With new technologies for diagnosis, studies on the therapeutic efficacy of natural products are always needed. Therefore, these areas are also included in the issue.

In this special issue, the publications have been included as described above, discussing various aspects of quality control of natural products in detail. More than hundreds of references totally found at the end of each publication will make this thematic issue useful not only for natural product researchers, but also for many scientists working in numerous fields. Much effort has been invested by all the contributing authors, reviewers, editors, and staff to have current information. Without their efforts and input, this issue could not exist. The editors sincerely appreciate the time spent and the knowledge shared in bringing this issue to fruition.

*Ying-Yong Zhao* *Ying-Yong Zhao* *Shuang-Qing Zhang* *Shuang-Qing Zhang* *Feng Wei* *Feng Wei* *Yu-Ming Fan* *Yu-Ming Fan* *Feng Sun* *Feng Sun* *Shuhua Bai* *Shuhua Bai*
